# A Scoping Review of the Management of Benign Prostate Hyperplasia in Africa

**DOI:** 10.7759/cureus.31135

**Published:** 2022-11-05

**Authors:** Abdulahi Zubair, Samuel Davis, Damilola I Balogun, Emeka Nwokeocha, Chineme-anyaeze Chiedozie, Damilola Jesuyajolu

**Affiliations:** 1 Urology, Surgery Interest Group of Africa, Lagos, NGA; 2 Neurosurgery, Association of Future African Neurosurgeons, Yaoundé, CMR

**Keywords:** prostate, benign prostate hyperplasia, turp, open prostatectomy, holep, surgical, medical, africa, urology, bph management

## Abstract

Benign prostatic hyperplasia (BPH) is a non-malignant prostate gland enlargement of unknown cause that affects more than 50% of men over 60 and is the most common cause of bladder outlet obstruction and voiding symptoms. BPH is treated primarily with watchful waiting, phytotherapy (herbs), and medical or surgical options. In this study, we sought to examine the different management practices in African urological centers, outcomes of management, and complications. A literature search was conducted using PubMed, African Journal Online, and Google Scholar regarding the management of BPH from inception till date. Articles were selected based on their relevance to the management of benign prostatic enlargement in Africa. Results are reported according to the Preferred Reporting Items for Systematic Reviews and Meta-Analyses (PRISMA) extension for scoping reviews. The studies included were conducted from 1997 to 2022. They were from eight different African countries (Nigeria, Kenya, Togo, Ethiopia, Egypt, South Africa, Ghana, and Congo), with Nigeria contributing the most with 10 studies. Exactly 2999 patients were included in the study. Seventy-three (73.49%) percent of these patients totaling 2204, underwent surgical management of BPH, 124 (4.13%) patients were treated with phytomedicines or herbs, and 684 (22.80%) patients were treated with medical therapy. The complications and outcomes were studied and collated. A total of 808 patients opted for non-surgical treatment for BPH in the included studies. In this group, 124 were treated using phytochemicals or natural herbs, and 648 were treated with standard prescription medications. While surgical treatment for benign prostatic enlargement is shifting towards minimally invasive procedures in the developed world, open prostatectomy is still quite popular in Africa. Further research should focus not only on the reason for these disparities in management but also on the rationale for the selection of medical, surgical, or phytotherapy in African urological centres.

## Introduction and background

Benign prostatic hyperplasia (BPH) is the benign growth of the prostate, usually seen in aging male adults. This enlargement is associated with bladder outflow obstruction, resulting in presentations in urology outpatient clinics or ERs with lower urinary tract symptoms (LUTS) such as urgency, frequency, nocturia, incomplete urination, and a weak urinary stream. Its prevalence increases with age; autopsy findings showed it is as high as 50-60% for males in their 60s, increasing to 80-90% for those over 70 years of age [1,2].

BPH is uncommon in people less than 40 years of age and more common in people with a family history of the condition, obesity, and metabolic syndrome [3-5]. The American Urological Association and the European Association of Urology guidelines advise that clinicians should obtain a history of symptoms, perform a physical examination, obtain urine for urinalysis, and use a symptom scoring method (e.g., the International Prostate Symptom Score) in the initial evaluation of patients with LUTS. Furthermore, patients should be counseled on options for intervention, which can include watchful waiting, behavioral or lifestyle modifications, medical or pharmacotherapy, phytotherapy, surgery, or a combination of these methods [6,7].

There are medical and surgical approaches for treating BPH. Medical management of BPH is often first-line, especially after the diagnosis is first made [8]. Monotherapy or combination therapy with alpha-blockers (e.g., alfuzosin, doxazosin, tamsulosin), 5-alpha reductase inhibitors (e.g., finasteride and dutasteride), and other medications (e.g., antimuscarinics) are typically used. In addition, a re-evaluation of the patient is indicated, with a need to reconsider their international prostate symptom score (IPSS) and to have uroflowmetry and post-void residual investigations done [6].

Surgical management of BPH includes open prostatectomy (transvesical, laparoscopic, or robotic-assisted prostatectomy), transurethral holmium laser ablation of the prostate, transurethral holmium laser enucleation of the prostate, holmium laser resection of the prostate (HoLEP), transurethral microwave therapy (TUMT), photoselective vaporization of the prostate (PVP), greenlight laser therapy, thulium laser therapy, prostatic urethral lift (PUL), transurethral incision of the prostate (TUIP), transurethral vaporization of the prostate (TUVP), and transurethral resection of the prostate (TURP). TURP is a minimally invasive endourological procedure that employs a monopolar or bipolar current-based resection of the prostate and is widely regarded as the current gold standard for the management of BPH [9,10].

Patients with bothersome LUTS/BPH who elect initial medical management and do not have symptom improvement and/or experience intolerable side effects should undergo further evaluation and consideration of a change in medical management or surgical intervention [6]. Other indications for surgical intervention are recurrent acute urinary retention, recurrent urinary tract infection, recurrent bladder stones, failed voiding trials, recurrent gross haematuria and renal insufficiency secondary to obstruction, a desire to terminate medical therapy, and financial constraints associated with medical therapy [11,12]. Despite advances in the treatment of BPH in the developed world, with propositions for future therapies like novel drug therapies, and surgical approaches, there has been little to no change in the management practices in Africa, especially in Sub-Saharan Africa [4]. Several factors, such as the dearth of equipment, skilled manpower, and power supply, have been put forward as the stumbling blocks to these modern interventions [12-15].

This study examines the common treatment modalities for BPH across the continent of Africa, looking at medical, surgical, and phytotherapy or herbal interventions, as well as the different options within these broad classifications. We also sought to examine the intricacies of these broad treatment options and outcomes.

## Review

Methods

The Preferred Reporting Items for Systematic Reviews and Meta-Analyses extension for Scoping Reviews (PRISMA-ScR) checklist was used in conducting this study. A literature search was conducted using PubMed, African Journal Online, and Google Scholar, limited to articles published in English, regarding the management of BPH from inception till date. Articles were selected based on their relevance to managing benign prostatic enlargement in Africa. To limit bias, two reviewers working in pairs screened titles and abstracts; then screened full-text studies for inclusion, with any lack of consensus discussed with a third reviewer. A data chart was used to extract relevant data from the included studies. Reviews, meta-analyses, abstracts, conference presentations, commentaries, case reports, and letters to the editors were excluded. Data on the treatment choice, outcome and subtype of medical or surgical management options were pooled together. Twenty-one studies fully satisfied the eligibility criteria and were used in this review. The search strategy was jointly devised by the authors and is summarized below. The final search results were exported into Rayyan.ai (a systematic review software) for the removal of duplicates and the rest of the screening.

Titles and abstracts were screened first, emphasizing articles that specified BPH in African countries, while a full-text screening was followed by a critical appraisal of the management modality used. Next, relevant data were extracted from the identified studies. The data included: the first author; the title of the study; year of publication; country of origin; sample size; mean age; method of diagnosis; the type of medical intervention (medical, surgical, or phytotherapy/herbs), the subtype of medical intervention; the subtype of surgical intervention; outcomes of care (e.g., complications and rates), and follow-up period. This is presented in Table 1 below.

**Table 1:** Search strategy.

Database	Search	Papers
PubMed	(Benign prostate hyperplasia) AND (Africa) ("management"[MeSH Terms] OR "treatment"[All Fields] OR ("benign prostate enlargement) (Africa) (management) (treatment), benign prostate hypertrophy) (Africa) (treatment) (management)	523
Google Scholar	Benign prostate hyperplasia/hypertrophy/enlargement and Africa	The first 100 pages were searched. No additional studies seen.
African Journal Online	Benign prostate hyperplasia	45 results were seen. No additional study was identified.

Scope of the Review

**Figure 1:**
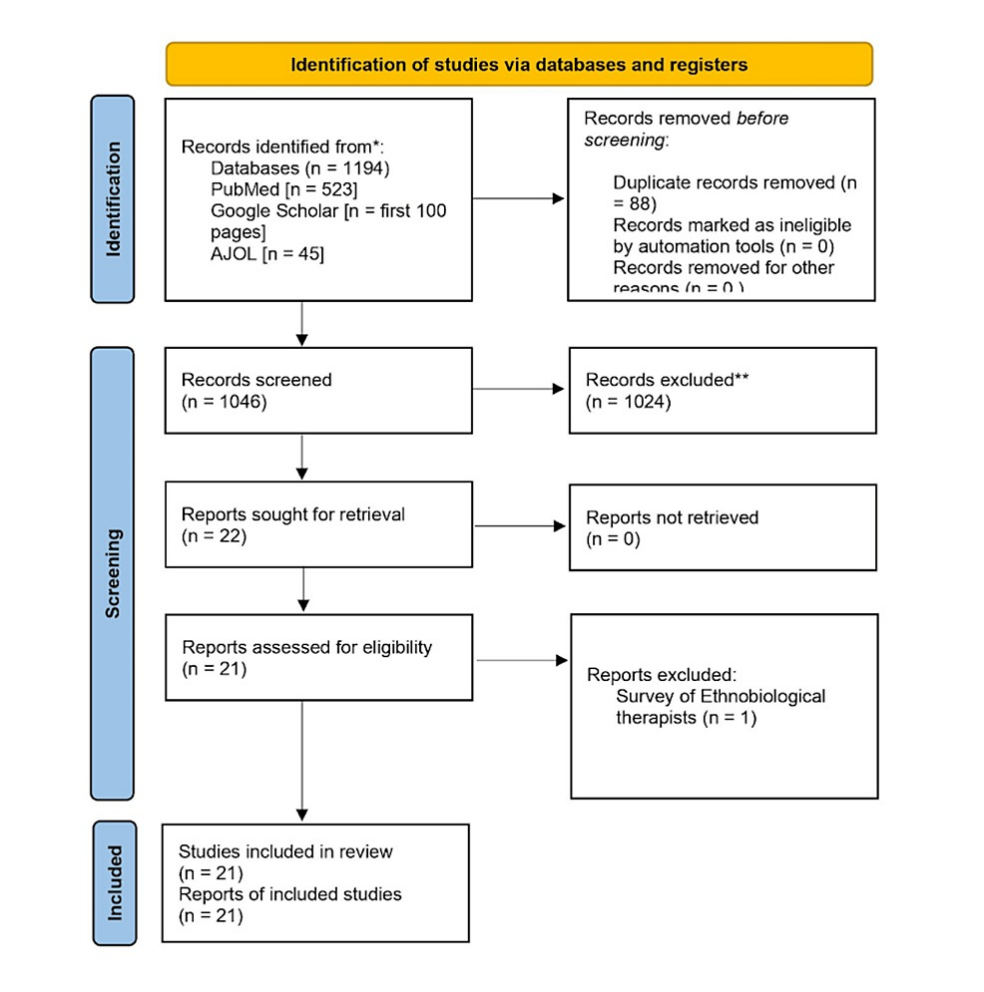
PRISMA flowchart. PRISMA: Preferred Reporting Items for Systematic Reviews and Meta-Analyses; AJOL: African Journal Online.

We reviewed all pooled papers and scrutinized them following the inclusion and exclusion criteria, as previously agreed by the authors. Duplicates were immediately removed before the screening process, and subsequently, the studies relating to the management of BPH in Africa were collated for further review and screening. This is shown below in Figure 1. 

Total papers included after duplicates were removed: 1194; Papers included after the screening process: 21.

Results

The studies were conducted from 1997 to 2022 from eight different African countries (Nigeria, Kenya, Togo, Ethiopia, Egypt, South Africa, Ghana, and Congo; Nigeria contributed the most with 10 studies). A total of 2999 patients were included in the study. Table 2 details the countries of origin of the studies included in this paper, while Table 3 presents the main characteristics of each study we reviewed. 

**Table 2:** Country of origin for the studies.

Country of origin	Studies
Nigeria	10
Kenya	3
Togo	1
Ethiopia	2
South Africa	1
Egypt	2
Ghana	1
Congo	2

**Table 3:** Study characteristics.

Author [ref.]	Year of Publication	Country of Origin	Sample Size
Ofoha CG et al. [10]	2021	Nigeria	59
Salako AA et al. [14]	2016	Nigeria	268
Olapade EO et al. [16]	2001	Nigeria	52
Ahmed Gadam I et al. [12]	2012	Nigeria	253
Kiptoon DK et al. [17]	2004	Kenya	85
Odusanya BO [18]	2015	Nigeria	90
Abdullahi M, Aji SA [19]	2016	Nigeria	72
Kpatcha TM et al. [20]	2016	Togo	54
Oliech JS [21]	2007	Kenya	550
Ugwumba FO et al. [13]	2014	Nigeria	297
Deneke A et al. [22]	2020	Ethiopia	89
Elshal AM et al. [23]	2016	Egypt	183
Alemu MH [24]	2009	Ethiopia	71
Alhasan SU et al. [25]	2009	Nigeria	502
Ogbonna BC et al. [26]	1997	Nigeria	31
Hill AG and Njoroge P [27]	2002	Kenya	106
Steele GS, Sleep DJ [28]	1997	South Africa	47
Kyei MY et al. [29]	2012	Ghana	58
Sikangula I et al.	2007	Congo	32
Undie CU et al. [30]	2021	Nigeria	40
Fayad AS et al. [31]	2011	Egypt	60

Overall, 2204 underwent surgical management of BPH. This represents around 73.49% of all patients who had treatment for BPH in this study, making surgery the most common management approach for BPH in this study. This is shown in Table 4 below. Surgery was offered as the initial management in some instances or following failed medical treatment [21,25]. A total of 124 patients were treated with phytomedicines or herbs, with varying degrees of success. In all, 684 patients were treated with medical therapy, seemingly as the first-line or to buy time before surgery [21]. Around 13 patients who had medical therapy underwent surgery in the same study [25]. Amongst all patients who had surgery done, 734 patients had TURP. This represents 33.03% of all cases. Varying complications with TURP are described in Table 5 in more detail. In these studies, UTI (7.34%) and clot retention (6.67%) were the most common complications of TURP. Other complications include epididymo-orchitis (3.01%), incontinence (0.56%), bladder neck stenosis (3.33%), urethral stricture (3.33%), hemorrhage (1.08%), and a need for reoperation (1.07%). Additionally, 1261 patients, representing 57.21% of all patients who had surgery, underwent open prostatectomy. Table 6 provides the details of all the open prostatectomy cases. Transvesical prostatectomy, with 73.8%, is by far the most common method of all open prostatectomies (Table 7). In contrast, retropubic prostatectomy was done in 255 of all patients in this study, representing 21.75% of all open prostatectomies. Only 47 patients were treated with transurethral needle ablation (TUNA).

**Table 4:** Overview of all surgical cases. TURP: Transurethral resection of the prostate; HOLeP: Holmium laser resection of the prostate; TUNA: Transurethral needle ablation.

Procedure	Open prostatectomy	TURP	HOLeP	TUNA
Number of Patients	1261	734	162	47

**Table 5:** A breakdown of TURP cases. TURP: Transurethral resection of the prostate.

Author	Sample Size
Ofoha CG et al.	30
Salako AA et al.	21
DKiptoon DK et al.	16
Deneke A et al.	58
Alemu MH	65
Alhasan SU et al.	502
Kyei MY et al.	12
Fayad AS et al.	30
Total	734

**Table 6:** A breakdown of open prostatectomy cases. Sources: [10, 14, 12, 17, 20, 13, 22, 23, 24, 27, 29]

Author	Open prostatectomy
Ofoha CG et al.	29
Salako AA et al.	247
Ahmed Gadam I et al.	253
Kiptoon DK et al.	69
Kpatcha TM et al.	54
Ugwumba FO et al.	297
Deneke A et al.	31
Elshal AM et al.	91
Alemu MH	6
Hill AG and Njoroge P	106
Kyei MY et al.	46
Sikangula I et al.	32
Total	1261

**Table 7:** Subtypes of open prostatectomy.

Open prostatectomy	Sample size
Retropubic prostatectomy	255
Transvesical prostatectomy	865
Retropubic prostatectomy with inguinal herniorrhaphy	36
Transvesical prostatectomy and cystolithotomy	7
Transvesical prostatectomy and inguinal herniorrhaphy	5
Transvesical prostatectomy and diverticulectomy	3
Retropubic prostatectomy and hydrocelectomy	1
Total	1172

Overall, 162 patients (seen in Table 8) had HoLeP in the studies reviewed, representing 7.35% of all surgical cases in this review. The commonest complications reported are hemorrhage requiring transfusion 10%, clot retention, and epididymo-orchitis 0.76%, respectively.

**Table 8:** Breakdown of HoLeP cases. Source: [23], [30], [31]. HOLeP: Holmium laser resection of the prostate.

Author	HOLEP/Laser enucleation
Elshal AM et al.	92
Undie CU et al.	40
Fayad AS et al.	30
Total	162

Approximately 47 people were treated with TUNA representing 2.13% of patients that had surgery; all the patients had haematuria. The treatment failed in six people, and one patient developed acute epididymo-orchitis. This is represented in Table 9 below. Overall, the rates of complications from the different surgical approaches (i.e., TURP, TUNA, OP, and HoLeP) were collated, and this has been presented in Table 10. 

**Table 9:** A breakdown of TUNA cases. Source: [28]. TUNA: Transurethral needle ablation.

Author	Sample Size
Steele GS, Sleep DJ	47

**Table 10:** Rates of complication of surgical approaches.

Method	Open Prostatectomy	TURP	HOLeP	TUNA
Clot retention	7.27%	6.67%	0.76%	
Epidydymoorchitis	4.19%	3.01%	0.76%	2.12
Post-op incontinence	6.58%	0.56%	0%	
Bladder neck stenosis	3.62%	3.33%	10.00%	
Urethral stricture	2.57%	3.33%	0%	
Haemorrhage	11.14%	1.13%	10.61%	
Infection	12.81%	7.34%	0%	
Reoperation	4.81%	1.07%	2.50%	12.76

On the other hand, a total of 808 patients opted for non-surgical treatment for BPH in the studies reviewed. In this group, 124 were treated using phytochemicals or herbs, and 648 were treated with standard prescription medications. In the latter group, 74 were treated with alpha-blockers, 30 with five alpha-reductase inhibitors, and 30 with a combination of an alpha-blocker and a five alpha-reductase inhibitor. In contrast, the remaining 550 were treated with standard medical treatment that was not specified. Out of the 124 patients treated with phytochemicals or herbs, 77.4% were said to have had successful outcomes (i.e., an improvement in LUTS). The commonest herbal preparation or phytochemical used in cited studies is Prostacare, a saw palmetto extract [19]. For the group that was treated with standard medical therapy for BPH, about 72.4% experienced an improvement in symptoms in the time they were followed up, while 27.6% either experienced no improvement, experienced worsening of their symptoms, experienced side effects (hypotension) warranting conversation to surgical therapy or were lost to follow up. The diagnosis of BPH took the form of clinical reviews, including history taking and a physical examination. Around 71.2% of studies reviewed reported the use of prostate-specific antigen (PSA) monitoring in addition to the clinical reviews, 52.3% used IPSS, and 52.3% used TRUSS in addition to the clinical reviews. Only 42.85% of studies used PSA and IPSS, while 33.33% of studies used PSA, IPSS, and TRUSS. 

Discussion

In this study, we sought to examine the different prevalent management practices in African urological centers, outcomes of management, and complications. This review demonstrated that surgical management of BPH, representing around 73.49% of all the treatment approaches, is the most common approach to managing BPH in African urological centers. Amongst the surgical options for managing BPH, open prostatectomy was the most commonly used surgical approach, representing 57.21% of all surgeries, with TURP (33.03%), HoLeP (7.35%), and TUNA (2.13%) also being described. In contrast, minimally invasive procedures such as TURP, TUNA, Aquablation, and HoLeP have gained popularity in other parts of the world and are included in the guidelines of management [32]. This may be attributed to a shortage of endoscopic equipment and newer technologies; inadequately trained personnel in their usage; delayed presentation resulting in higher prostatic volumes at initial examination and surgical planning; and a steeper learning curve for some of the newer technologies [25,31,33]. In addition, varying methods of reporting complications and outcomes were encountered, demonstrating the need for adopting a unified system of reporting outcomes, like the Clavien-Dindo Grade, for reporting complications, as suggested by Oranusi CK et al. [34]. 

Results of this study showed that open prostatectomy had the highest rates of complications among all surgical options, while HoLeP had the least rates of complications in comparison with TURP. This is similar to the findings of Yin L et al., as HoLeP was shown to have fewer rates [35]. Complications reported in this study included UTI, clot retention, epididymo-orchitis, post-op incontinence, bladder neck stenosis, urethral stricture, hemorrhage, and a need for reoperation.

The non-surgical management of BPH was revealed to have been in the form of medical or pharmacotherapy and phytotherapy, with the former being more prevalent in the studies examined. Six hundred eighty-four patients were treated with medical therapy, mostly with alpha-blockers as a monotherapy or in combination with other drug classes. Five alpha-reductase inhibitors were also employed in the medical therapy for BPH. This is similar to the recommendations of the American Urological Association [6]. For the group that was treated with standard medical therapy for BPH, about 72.4% experienced an improvement in symptoms similar to Roehrborn CG and Rosen RC [36]. A total of 124 patients were treated with phytochemicals, with a significant proportion showing positive outcomes. However, these were not captured using established symptom-scoring modalities or quality-of-life assessments in all the cases described. Nevertheless, 77.4% were said to have had successful outcomes (i.e., an improvement in LUTS), and phytochemicals are effective to some degree, warranting their inclusion in different guidelines for managing BPH. The commonest herbal preparation or phytochemical used in cited studies is Prostacare, a saw palmetto extract [19]. In contrast, pumpkin (Cucurbita pepo) is the most common phytochemical used in the management of BPH across the world [37].

The diagnosis of BPH was made after history-taking and a physical examination (a digital rectal exam). Around 71.2% of studies reviewed reported the use of PSA monitoring in addition to the clinical reviews, 52.3% used IPSS, and 52.3% used TRUSS in addition to the clinical reviews. Only 42.85% of studies used PSA and IPSS, while 33.33% of studies used PSA, IPSS, and TRUSS. As per the United Kingdom guidelines, multiparametric MRI should be the gold standard for determining prostatic volume, but we have yet to find any mention of this in the studies reviewed [38]. International guidelines also recommend uroflowmetry, post-void residual (PVR), and MRI or CT. However, we found no mention of these in the peri-operative review of the patients in this study. Ojewola RW et al. [39] proposed the use of uroflowmetry in addition to clinical history, digital rectal exam, IPSS, and an ultrasound scan of the patient. Thus, there is a need to establish nationally or continentally approved guidelines for diagnosing BPH in Africa. 

The amount of existing research on this subject is exhaustive, and many scoping reviews, systematic reviews, and meta-analyses have provided evidence on this topic. This study, however, to the best of our knowledge, is the first to look exclusively at the African literature to generate evidence that may guide local practices. More studies may need to be done on a geographic basis to generate locally relevant guidelines and practices that will improve patient care. Despite the comprehensive nature of this study, we had some limitations. First, the small sample size and the few African studies covered in this review make generalization difficult. Because we included articles only in English and searched four databases, we may have missed potentially relevant papers from non-anglophone countries. Finally, the non-disclosure of some of the papers on specifics of management, a standardized method of measuring outcome (e.g., IPSS vs. QOL) means that there are limitations to the outcome analysis. 

## Conclusions

While surgical treatment for benign prostatic enlargement is shifting towards minimally invasive procedures in the developed world, open prostatectomy is still quite popular in Africa. In most situations, medical or pharmacotherapy is the first line of treatment; however, surgical intervention and phytotherapy/herbal remedies are also highly popular. Typically, a diagnosis is made based on a patient's medical history and physical examination; however, symptom-scoring questionnaires and more complex diagnostic techniques are not widely used at this time. Therefore, further research should focus not only on the reasons for these disparities in management but also on the rationale for the selection of medical, surgical, or phytotherapy in African urological centres.
